# Gun Shot Wound, Death, Autopsy

**Published:** 1857-06

**Authors:** Geo. Shedd

**Affiliations:** Denmark, Iowa


### IOWA MEDICAL JOURNAL.

VOL. IV. KEOKUK, IOWA, MAY AND JUNE, 1857. NO. 1.

### ORIGINAL COMMUNICATIONS.

Surgeon Genl's Office LIBRARY. 14700 Washington, D.C.

### GUN SHOT WOUND. DEATH. AUTOPSY.

REPORTED BY GEO. SHEDD, M. D., DENMARK, IOWA.

In the twilight of the evening of the 13th of November last, two men, each ignorant of the presence of the other in the timber, were hunting deer upon the bottom of the Skunk, north of Denmark, when one, a Mr. Stetson Ralf, of Washington township, while bending forward under a bush, himself trying to espy a deer, was mistaken by the other for that animal, and shot.

Presuming this case, unusual, I apprehend, in many particulars, may afford some interest, and perhaps a subject of thought to some of the readers of the "Journal," I send you the following brief history of it from my first examination on that fatal evening, to its fatal termination, nine days afterwards, together with the result of an examination had twelve hours after death.

The ball was found to have entered his right side at about its greatest curvature and over the floating ribs, and to have passed in the direction to meet the lumbar vertebræ. The ball was still in his body, but *where*, I could not ascertain. The lower extremeties were palsied, and he was suffering very little from the injury.

14th, 10 o'clock, A. M.—Found the patient quite free from pain and fever, but perfectly paralysed below and including his iliac region. Sensation however seemed but little impaired in the upper portions of his body. Had rested some during the night. Complained of some tenderness across the upper and middle portions of his bowels upon pressure. I passed a catheter into his bladder, and drew off four teacups full of dark, bloody urine, much to his relief. Had vomited some clotted blood and bloody matter during the night. I ordered cloths wet in tepid water laid on his bowels and around him as far as practicable, and changed as soon and as often as they should become heated.

Evening—Much the same as in the morning. Drew urine freely, but less bloody than at the former drawing.

15th, 10 o'clock, A. M—Had had a comfortable night—slept considerably. Pulse 80. Urine quite free and healthy in appearance. Had more appetite than was prudent to indulge. Took gruel sparingly.

17th.—Still comfortable, but complained of some tenderness in the back and left side, if pressed. Little or no pain. Rested well the previous day and night. Appetite quite sufficient. Urine still normal. Pulse still about 80, and normal. Some bloody expectoration. Tenderness over the stomach and bowels seemed rather abating, and sensation seemed a little improving—he thought he could feel pressure upon the upper and front part of his thighs. I cleansed out the rectum with injections.

18th, 5 P. M.—Was more restless in the latter part of the night—increased heat and pain in back and left side, but cloths dipped in cooler water and more frequently applied had afforded relief. Pulse slightly increased in frequency. No cough, and no pain, or but slight, complained of anywhere. Some jaundice appeared to-day upon the surface of the skin, and some fatigue was felt from talking.

19th, 2 P. M.—Much as at the evening previous, except the urine had become bloody again, though still free, and the jaundice was increasing. Moved his bowels by injections. Could lie and rest upon his side quite comfortably.

20th, 2 P. M.—Summoned to see him—was worse. Bladder greatly distended and very tender, and nurse could draw but very little urine. A large instrument drew away one teacup full, mixed with blood, and exceedingly fetid and pungent; some temporary relief. Suspected large coagula of blood in the bladder. Increased tenderness over the whole bowels where sensation remained, but no portion much tumefied except the bladder. Great restlessness and depression of countenance and spirits; complained that the room felt cold but was not chilly. I diagnosed soon peritonial inflammation from the gun shot wound. No cough or pectoral pain, but some dullness on percussion on the right lung. Pulse 120, small and feeble.

21st.—Intense and protracted chill about midnight, and another about noon to-day. Involuntary discharges from bowels and probably slightly from the bladder, with most sickening fetor. Pulse scarcely perceptible—counted 130. Disease obviously extending and life fast ebbing out. Clammy sweat after the first chill.

22d.—Death seemed to commence about 2 o'clock in the morning, but life was not extinct till 9 o'clock, P. M., full 18 hours in dying.

Such is a brief, but, I believe, accurate history of the case, gathered from notes taken from time to time, by myself, and carefully compared with the observations of nurses and friends, the day after the funeral.

### AUTOPSY.

In this I was assisted by Dr. Whinnery, of Fort Madison. On laying open the abdominal parietes, an unusual sight met our view. The entire peritoneal lining of the parietes, the peritoneal coat of the stomach, bowels and bladder, in fact al upon which the eye could rest, except perhaps parts of the liver, presented gangrenous appearances; that is, those grayish, dark, purple and livid colors, that are usually regarded as concomitants of gangrene. Some of the parts were quite as dark as pale ink. The duodenum, transverse colon, the adjacent small bowels and caput coli together with parts upon the adjacent walls, were especially so. Several parts of the bowels that were laid open, showed a similar internal condition, with coats very much thickened. The omentum magnum was literally a mass of disease. The stomach and bowels were much distended; the bladder enormously so. Besides filling the pelvic cavity, the bladder presented a body above the pelvic brim as large as a three pint or a two quart cup. It was adherent to the peritoneum through most of its anterior surface, and laterally throughout the pelvis. Much putrid fetor was present, but not much serous effusion.

The parts were dissected out carefully and separately, and examined as far and as minutely as time permitted.

The liver was inflamed in parts and somewhat enlarged; the spleen was quite large.

The diaphragm on the right side, especially parts near the spine, was exceedingly inflamed and gangrenous in appearance. Some parts of it seemed to be a mass of clotted blood. The pericardium contained about two ounces of fluid, but was not particularly inflamed. Both auricles of the heart were inflamed and softened, while the left one was lined throughout with a tough, thick membrane of fibrine, which extended also into the pulmonary veins, but how far was not ascertained.

The right lung from its apex to the base was adherent in every part wherever its body could touch a plural surface, except a space of perhaps the length and breadth of a man's finger, opposite to the division between the upper and middle lobes of the lung. The two upper lobes were partially adherent, the two lower entirely so. These adhesions were all very intense. The whole right lung was hepatised, and most of the surfaces of the two lower lobes, presented the appearance of clotted blood. This appearance extended about one-fourth or one-third of an inch from the surface into the substance of the lung.

The left lung was healthy, and served the patient well to the end. It was about the only healthy spot found—a beautiful oasis in the midst of an arid desert and frightful desolation.

The right kidney was found pierced by the ball, in its upper portion and badly lacerated. It was a mass of clotted blood and inflammation of a dark gangrenous appearance.

The ball was found to have passed directly across the body, and through the processes of the lumbar vertebrae; a little posterior, it was thought, to the cord and yet barely so, thence by the left lateral process, it was turned posteriorly between the floating ribs of the left side, thence upwards following some of the larger muscles of the back or side. It was not found. It was the belief of the patient that the ball was lodged in the left side, in the region of the shoulder blade.

Here the examination was arrested, the funeral cortege having already assembled.

It may be worthy of remark that, at the examination, the corpse was the most deeply jaundiced of any the writer has ever seen. Dr. W., thinks he has seen one other equally so, a corpse deceased of yellow fever. Whatever cavity was laid open, except it bore a livid appearance, and whatever flesh was cut, the same jaundiced condition was manifested, as on the surface. The pericardium, the auricles of the heart, and the pseudo membrane found therein, were strikingly so.

And now several interesting pathological questions present themselves. Whence so much effusion of blood in the diaphragm and the two lower lobes of the right lung? The nearest of these parts, the diaphragm, is one and a half or two inches from the path of the ball, while some parts of the lung thus affected, are several inches remote, and are separated from the abdominal cavity by the wall of the diaphragm

Was this effusion the result of sympathy? Or will contact, or intimate connection, or proximity explain it?

It is the doctrine of the books, that the serous membranes when inflamed, pour out lymph and serum, and are not liable to gangrene, except they receive it from other and adjacent tissues. Now as stated in the autopsy, very little serum or other effusion was found in the abdominal cavity, and the whole peritoneal covering of the abdominal viscera, (save the glandular organs,) and much of the lining of the parieties, presented a dark, gray, livid and black appearance. Was this gangrene? if not, what was it, and whence this condition of the parts? Did the serous coat receive its discoloration from a sphacelated condition of the adjacent tissues? But there was no evidence from first to last, that those tissues were primarily affected. Perhaps, also under the circumstances, there is no conclusive evidence that they were not.

There was necessarily a paralysis of the part supplied with nerves from and below the lumbar region, but not necessarily so above, and no paralysis or diminution even of the powers of sensation had been discovered in the *surface* of the upper portions of the body. The *cavity* of the chest, as well as its surface, is supplied with nerves of sensation that arise from parts far above the injury. Why was there such an absence of rational symptons of disease in the cavity of the thorax, where disease was making such direful havoc? No cough, or disposition to cough was observed, no pain was complained of. Will sympathy with the paralysed parts below, explain this singular paralysis of the internal organs of the chest? If it was the effect of the shock communicated to the spinal cord, why did it not affect more sensibly the surface of the chest and upper parts of the bowels, that receive their nerves through the dorsal vertebræ.

For the last two days, it seemed that life could not continue long, and yet, after death had obviously commenced its work, he was full 18 hours in dying. At the examination, there was seemingly disease enough and violent enough to kill several men in ordinary circumstances, in a moiety of the time it killed him. How could life continue so long in the midst of and preyed upon, by such an amount of direful disease? Was it not the absence of the pain and suffering usually endured in such cases, that prolonged his life to such a degree? In his case, the functions of organic life, having their nervous origins in parts far above the wound, and measurably independent of the spinal cord, remained measurably intact, and the man lived after his injury, much as the tree lives, and died as the tree dies that is smitten with the blight, eking out a feeble life, till all life is gone.

*Remarks.*—The report of this case, so well and so minutely given, presents some interesting features, and deserves, as the author desires, a brief examination in connexion.

To the first question propounded, it is difficult to reply satisfactorily. Inflammation results in gangrene more readily in some tissues than in others, for while the cellular tissue and skin may become sphacelated by a moderate degree of inflammation, the blood vessels, ligaments, and tendons, etc., are pertinaceous of their integrity. This is the case more particularly in ordinary forms of inflammation, but in low erysipelatous inflammations, we have often a destruction more extensive, in which lesion, the vessels may partake largely. A solution of continuity, it is believed, may occur with the blood vessels, in which case they would yield to the pressure of the column of blood, hence the effusion as found in the progress of the *post-obit* examination in this case.

In these remarks it will be seen that erysipelatous inflammation is believed by the writer of these remarks, to have occurred. The accident occurred on the 13th of November, after which time, to the 20th, his condition gave faint hopes of ultimate recovery. But at the last date, grave symptoms presented themselves, which were those of severe inflammation. On the 21st he was still worse, and on the 22d dissolution was slowly approaching. Disorganization is now going on and such the condition of the structures, and such the change produced in them, that fluids could not in this state have been forced by injection through the blood vessels. If there be a want of integrity in these and the onward currents of blood cannot pass through those portions entirely destroyed, it may be safely presumed that effusion would readily take place at the boundary of that portion or part destroyed.

In the absence of the proof that there is a peculiar or specific inflammation of a gangrenous nature we are sometimes at a loss to account for certain appearances which they present. Close observation, however, has shown that in inflammation and the resulting gangrenous condition of the sub-serous tissues with which the serous tissues are connected and closely associated; that these last will often present a sphacelated condition, because of the infiltration of blood. The inflammation *may* extend, and so violently attack the serous tissue because of its contact and connexion with adjacent structures, as to result in spachelus. This is an extension of inflammation through the vascular connexion with parts in immediate relation, but is not an inflammation *per se* of the serous tissue itself. Serum and lymph are the usual terminations of serous inflammations; the adhesions, between the portions of the pleura pulmonalis, in this case, go to show that lymph was poured out.

D. L. Mc.

